# The Central Role of Cytochrome P450 in Xenobiotic Metabolism—A Brief Review on a Fascinating Enzyme Family

**DOI:** 10.3390/jox11030007

**Published:** 2021-06-22

**Authors:** Francisco Esteves, José Rueff, Michel Kranendonk

**Affiliations:** Center for Toxicogenomics and Human Health (ToxOmics), Genetics, Oncology and Huma Toxicology, NOVA Medical School/Faculty of Medical Sciences, Universidade NOVA de Lisboa, 1169-056 Lisboa, Portugal; jose.rueff@nms.unl.pt (J.R.); michel.kranendonk@nms.unl.pt (M.K.)

**Keywords:** Cytochrome P450 (CYP), drug-metabolizing enzymes (DMEs), xenobiotic, metabolism, toxicology, carcinogens, adverse drug reactions (ADRs)

## Abstract

Human Cytochrome P450 (CYP) enzymes constitute a superfamily of membrane-bound hemoproteins that are responsible for the metabolism of a wide variety of clinically, physiologically, and toxicologically important compounds. These heme-thiolate monooxygenases play a pivotal role in the detoxification of xenobiotics, participating in the metabolism of many structurally diverge compounds. This short-review is intended to provide a summary on the major roles of CYPs in Phase I xenobiotic metabolism. The manuscript is focused on eight main topics that include the most relevant aspects of past and current CYP research. Initially, (I) a general overview of the main aspects of absorption, distribution, metabolism, and excretion (ADME) of xenobiotics are presented. This is followed by (II) a background overview on major achievements in the past of the CYP research field. (III) Classification and nomenclature of CYPs is briefly reviewed, followed by (IV) a summary description on CYP’s location and function in mammals. Subsequently, (V) the physiological relevance of CYP as the cornerstone of Phase I xenobiotic metabolism is highlighted, followed by (VI) reviewing both genetic determinants and (VI) nongenetic factors in CYP function and activity. The last topic of the review (VIII) is focused on the current challenges of the CYP research field.

## 1. Xenobiotics Disposition and Excretion

Humans are continuously exposed to a wide variety of chemicals. An important portion of these compounds is not essential for maintenance of normal homeostasis. They are neither nutrients nor intermediate metabolites, produced from nutritional metabolism. Drugs, environmental pollutants, cosmetics, and even components present in our diet, such as food additives, form this extended group of xenobiotics in general harmless, but potentially toxic [1,2,3,4]. In a human lifetime, one might be exposed to 1–3 million different foreign compounds, which can accumulate within a variety of different organs and tissues [4]. Storage of xenobiotics can function as either a protective mechanism or as a mean by which bioaccumulation can trigger toxic effects. This potential toxic route depends on the physiologic relationship between the storage depot and the target tissues for a specific toxicant [1,3].

Xenobiotics are metabolized and ultimately eliminated through the urine, bile, and feces, with minor routes through expiration and sweat. However, without effective detoxification and subsequent excretion, many compounds may reach toxic levels and interfere with cellular homeostasis, leading to cellular and tissue damage, with detrimental effects on health [1,2,4]. Harmful cellular and tissue concentrations can be prevented by direct elimination of xenobiotics, whose mechanisms are dependent on the physico-chemical proprieties of the compounds. Yet, the vast majority undergo biotransformation by complex metabolic mechanisms, resulting in the formation of numerous metabolites, some of which have the potential to cause unintendedly, more toxic effects, i.e., bioactivation [1,4,5,6]. Detoxification routes comprise enzymatic functionalization and/or conjugation reactions that facilitate elimination and excretion. All combined, these pathways act jointly to detoxify xenobiotics and remove them from cells and tissues [1,3,4,6,7,8,9]. In chemical toxicology, it is therefore of great interest to have comprehensive and integrated knowledge of in vivo xenobiotic metabolism, to understand, predict and prevent potential health hazards through bioavailability, bioaccumulation, or generation of harmful reactive metabolites, after chemical exposure.

**Figure 1:**
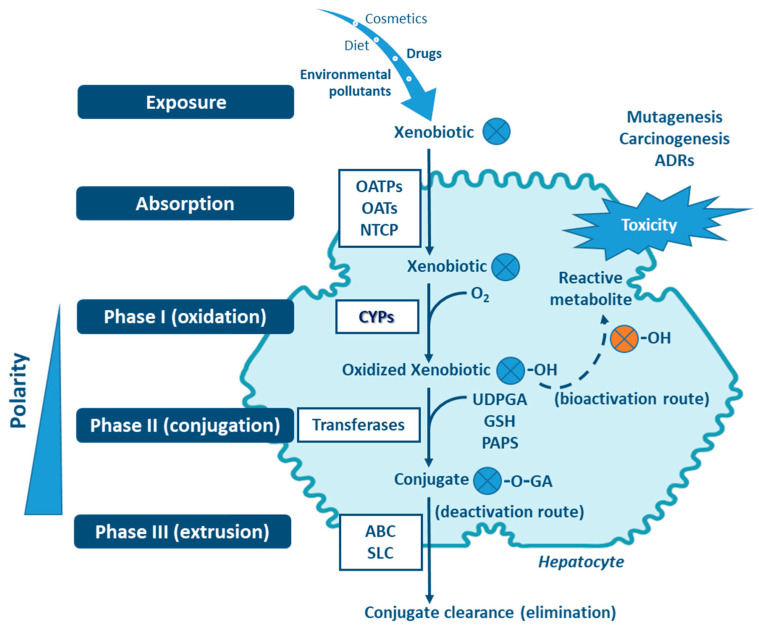
Xenobiotic metabolism in the hepatocyte and the central role of CYPs in biotransformation. Besides hydroxylation, exemplified here, CYPs catalyze a variety of other biotransformation reactions (e.g., epoxidation, dealkylation, oxygenation, dehydrogenation, dehalogenation, among others). Non-CYP mediated metabolism may also occur in Phase I via flavin-containing monooxygenases (FMOs), NAD(P)H:quinone oxidoreductases (NQOs), amine oxidases, alcohol dehydrogenases, esterases and peroxidases. ABC: ATP binding cassette (e.g., multidrug resistance protein family—MRP); GA: glucuronic acid; GSH: glutathione; NTCP: sodium taurocholate cotransporting polypeptide; OAT: organic anion transporters; OATP: organic anion transporting polypeptides; OH: hydroxyl; PAPS: phosphoadenosine-phosphosulfate; SLC: solute carrier transporters; UDPGA: uridine diphosphate-glucuronic acid. Transferases: glutathione S-transferases (GST), methyltransferases, glycine N-acyltransferase (GLYAT), N-acetyltransferases (NAT), sulfotransferases (SULT), UDP-glucuronosyltransferases (UGT).

Four stages can be distinguished in the processes of absortion, metabolism and cellular excretion of xenobiotics, namely (**i**) influx by transporter enzymes, biotransformation in (**ii**) Phases I and (**iii**) II, mediated by drug-metabolizing enzymes (DMEs), followed by (**iv**) Phase III, the excretion mediated mostly through transporter enzymes [1,6,9,10,11,12] (Figure 1). Organic anion transporting polypeptides (OATP), organic anion transporters (OAT) and sodium taurocholate cotransporting polypeptide (NTCP), are involved in the influx of the xenobiotics (reviewed in Murray M, Zhou F, 2017) [12]. Phase I enzymes, which include the cytochrome P450s (CYPs) superfamily—the major contributer— but also flavin-containing monooxygenases (FMOs), NAD(P)H:quinone oxidoreductases (NQOs), amine oxidases, alcohol dehydrogenases, esterases and peroxidases (reviewed in Gan J, et al., 2016) catalyze the oxidation, reduction or hydrolyses of primarily lipophilic xenobiotics into more polar molecules [7,13,14,15,16]. The introduction of polar groups by Phase I reactions provides sites that enable conjugation reactions, mediated by Phase II enzymes [5,7,17,18]. Although Phase II enzymes can directly act on the parent compound [19], typically Phase I-metabolites are conjugated with charged species, such as glucuronic acid, glutathione, sulfate, amino acids (glycine, taurine, glutamic acid), methyl or acetyl groups. Addition of these large anionic groups, which may detoxify reactive electrophiles (either parent compound or Phase I metabolite), produce Phase II metabolites, with increased hydrophilicity and molecular weight, which in larger part are not able to diffuse across phospholipid membrane barrier (reviewed in Jančová P, Šiller M, 2012) [6,10,11,13,19,20]. Phase III xenobiotic transporters excrete hydrophilic conjugates, with the anionic groups acting as affinity tags for a variety of membrane carriers belonging to two main clusters: ATP binding cassette (ABC), including the multidrug resistance protein (MRP) family, and solute carrier (SLC) transporters (reviewed in Döring B, Petzinger E, 2014) [21,22,23].

There are major inter- and intra-individual variations in the capacity to metabolize, detoxify and extrude xenobiotics (see below). These are of genetic, epigenetic, environmental, and physio- or pathophysiological origin, and vary during lifetime [9,13,21,24,25,26,27,28,29]. Most xenobiotics are detoxified and cleared through an intricate network of multiple enzymes and pathways. The relationship between xenobiotic local/cellular concentration, specific enzymes affinity, tissue specific enzyme expression, stability, and cofactors availability, often determine which metabolic reactions dominate in a given individual, at one precise moment [1,7,30].

## 2. Historical Aspects of Cytochrome P450s Research

Cytochrome P450s comprise the major Phase I family of enzymes capable of catalyzing the oxidative biotransformation of a vast majority of lipophilic xenobiotics and are the focus of research in areas such as clinical pharmacology and toxicology [5,18,25,31,32,33]. The early reports dealing with CYP, dated back to the 1940s and were related with in vitro studies on the metabolism of steroids and xenobiotics, including drugs and carcinogens [34].

The establishment of the CYP research field goes back to the late 50s/early 60s, when several groups focused on a particular pigment in animal liver microsomes, which seemed to be directly related with specific hepatic functions [35,36]. In 1962, a major breakthrough was obtained by Drs. Tsuneo Omura and Ryo Sato, describing for the first time, the spectral observation of CYP in liver microsomes [37]. In fact, the name of CYP (*P450*) is derived from the characteristic absorption maximum at 450 nm, observed in differential spectra of the reduced CO-bond enzyme complex. Initially, CYP was thought to be a single enzymatic entity, which was believed to exist almost exclusively in the liver, responsible for the metabolism of drugs and other foreign chemicals [38,39,40]. Several seminal reports described the inducibility of CYP activities, of clinical relevance in pharmacology and therapeutics [40,41,42]. Subsequently, other remarkable discoveries were made, namely: the role of CYPs in steroid and fatty acid hydroxylation, both in adrenal cortex and liver (1963) [43,44]; protein separation of CYPs [45] and their purification [46,47]; identification and biochemical characterization of multiple CYP forms—evidencing the existence of numerous isoenzymes (late 60s/early 70s) [39,47,48,49,50]; biophysical studies and biochemical characterization of bacterial CYP (P450_cam_ or CYP101A1)—of importance to establish template protein structures to be used in homology modeling (1968) [51]; and data supporting models on oxygen activation, evidencing a stepwise process involving C-H bond breaking (1978) [52]. In 1983, the first complete analysis of a CYP gene (rat) was accomplished [53]. Thereafter, the evolution of molecular genetic techniques led researchers to uncover the extensive inter- and intra-species variability of the CYP genes superfamily. Genetic studies have shown the considerable inter-individual differences in expression of CYP isoenzymes in the human population (since the 80s) [30,54,55,56,57,58,59]. In the waking of the new millennium, 57 human CYP genes (plus multiple pseudogenes) were identified by the human genome project (2003) [26,60,61]. During this period other significant contributions were the development of mammalian (including human) heterologous CYP expression, using recombinant DNA technology (early 90s)—critical for crystallography and functional studies [62,63,64,65]; application of engineered bacterial strains expressing human CYP enzymes complex in biotransformation and genotoxicity studies of xenobiotics (late 90s) [66,67]; the first CYP proteins structures solved (bacterial P450_cam_ and P450BM3) (early 80s and 90s) [68,69,70], followed by the crystal structures of human CYPs—particularly high-resolution structures (2000s) [71,72,73,74]; and the description of determinant protein-protein interactions within CYP enzymes complex—impacting CYP activity (2000s and early 10s) [75,76,77,78,79,80,81].

## 3. Classification and Nomenclature of Human Cytochrome P450s

Back in the 80s, studies enabled by the big boom in molecular biology demonstrated that CYP genes are ubiquitously present in almost all life forms, from prokaryotes to humans, adding a new dimension to the complex repertoire of functions catalyzed by this super enzyme family [53,54]. The genes encoding these heme-thiolate monooxygenases capable of catalyzing the oxidative biotransformation of endogenous compounds and xenobiotics, diverged from a single ancestor in an evolutionary process started 3 billion years ago [82,83]. In contrast with “conventional” enzymes, which normally demonstrate high substrate specificity and turnover rates, human CYP isoenzymes involved in drug metabolism, evolved favoring low substrate specificity and turnover rates, characteristics of DMEs in general [30].

**Figure 2:**
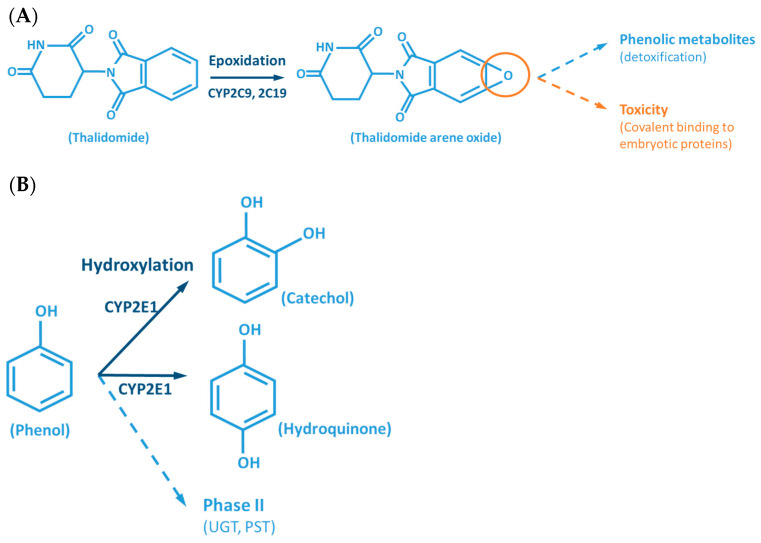

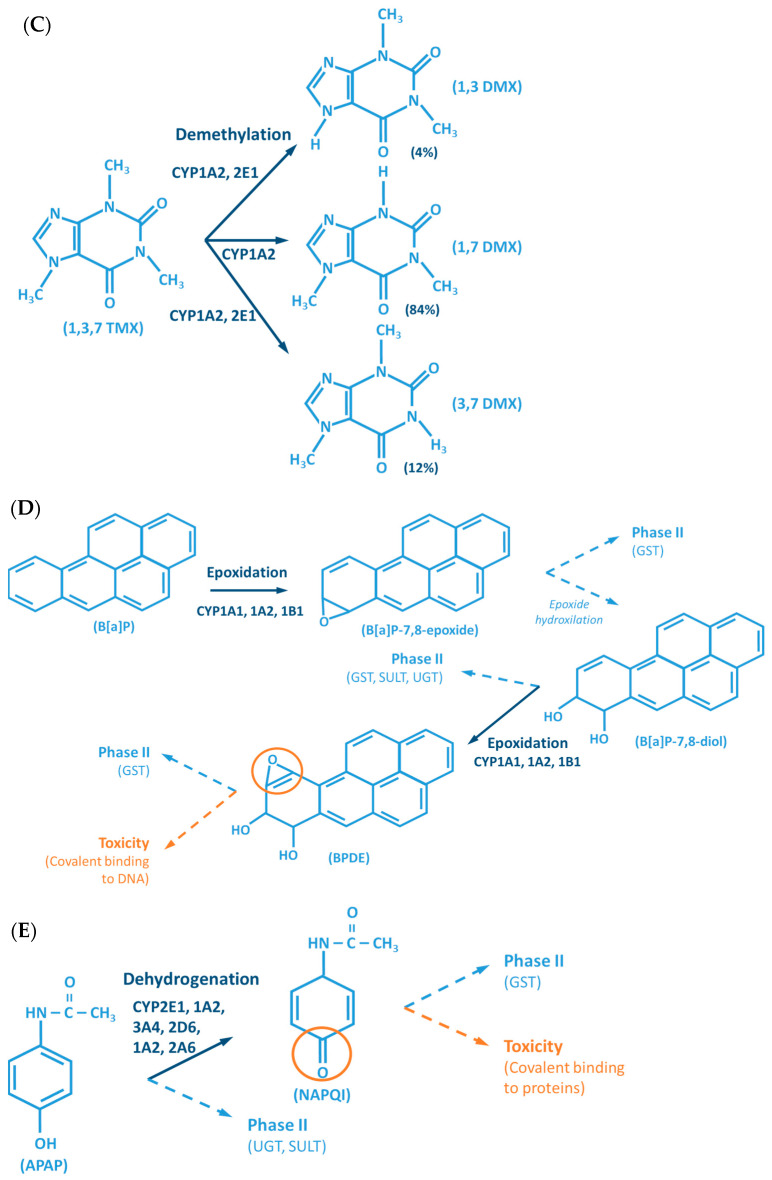

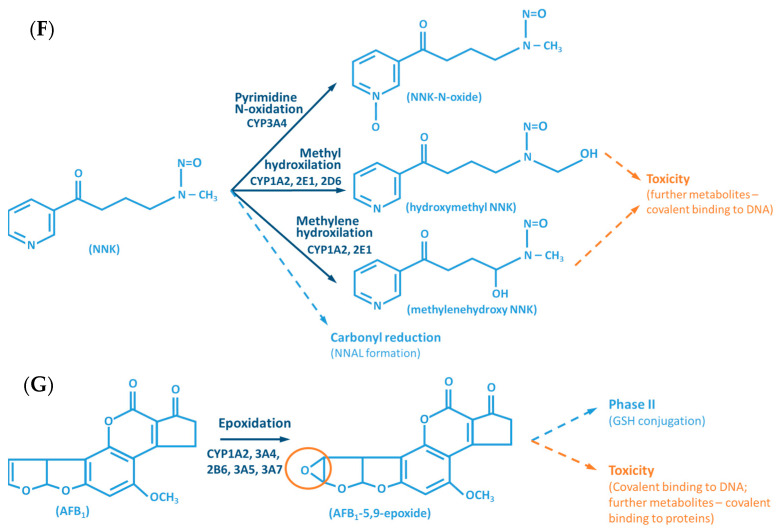
Examples of reactions catalyzed by CYPs. (**A**) CYP-mediated bioactivation of thalidomide. (**B**) CYP-mediated hydroxylation of phenol. (**C**) CYP-mediated metabolism of caffeine, with multiple metabolites. (**D**) Bioactivation of benzo[a]pyrene mediated by CYP enzymes. (**E**) CYP-mediated metabolism of paracetamol and its potential toxicity. (**F**) CYP-mediated bioactivation of NNK. (**G**) Bioactivation of aflatoxin B_1_ by CYP enzymes. AFB_1_: Aflatoxin B_1_. APAP: acetaminophen; B[a]P: benzo[a]pyrene; BPDE: benzo[a]pyrene diol epoxide; DMX: dimethylxanthine; GST: glutathione S-transferases; NAPQI: N-acetyl-p-benzoquinone imine; NNAL: 4-(methylnitrosamino)-1-(3-pyridyl)-1-butanol; NNK: nitrosamine 4-(methylnitrosamino)-1-(3-pyridyl)-1-butanone; PST: phenol sulfotransferase; SULT: sulfotransferases; TMX: trimethylxanthine; UGT: UDP-glucuronosyltransferases.

Although the outcome of the CYP-mediated metabolism prevents bioaccumulation by chemically transforming lipophilic compounds (readily absorbed) into hydrophilic metabolites (readily excreted) (Figure 2), CYP mediated biotransformation as well as other DMEs, evolved in a species-specific manner [54]. Historically, species of rodents, dogs, rabbits and pigs were considered to be suitable organisms for comparative drug metabolism studies. However, the variability in xenobiotic metabolism among species, in particular CYP metabolism, is evidenced by the fact that mutagenicity profiles of chemicals determined by the Ames test [84,85] when using human liver S9 fraction is significantly different from the ones observed with the rat liver S9 fraction [86]. This raises an important issue regarding the utility and reliability of results obtained from toxicological studies, using animal materials and models for risk assessments of human exposure [87]. Several factors must be carefully considered for the appropriate selection of an animal model which adequately mimics the human metabolism of a particular xenobiotic. Species-specific expression, regulation and function of CYPs have to be taken into account to avoid significant deviations. This inter-species variability is harshly exemplified by the case of thalidomide, in the late 50s/early 60s. This drug was commercialized as a non-addictive, non-barbiturate hypnotic and anti-emetic, and used for treatment of morning sickness of pregnant women. Thalidomide was tested for potential teratogenicity in mice and considered to be safe, however its use led to severe birth defects involving limb malformation. Teratogenicity is thought to occur through a reactive metabolite of thalidomide (arene oxide) (Figure 2A), formed in significantly higher proportions in human (and rabbit) liver than in mouse liver. After this occurrence, U.S. Food and Drug Administration (FDA), and medicine agencies worldwide, implemented obligatory full multi-species (in vivo) teratogenicity testing as a requisite for drug approval [88,89]. Another example is the metabolism and effect of aflatoxin B_1_ in trout. Aflatoxin B_1_, the most potent hepatic chemical carcinogen known today is activated via a CYP-dependent reaction (Figure 2G). Interestingly, and in contrast with the rat model, trout exposure to aflatoxin B_1_ may result in a hepatocellular carcinoma with relevant similarity to the one found in humans [90,91].

**Figure 3:**
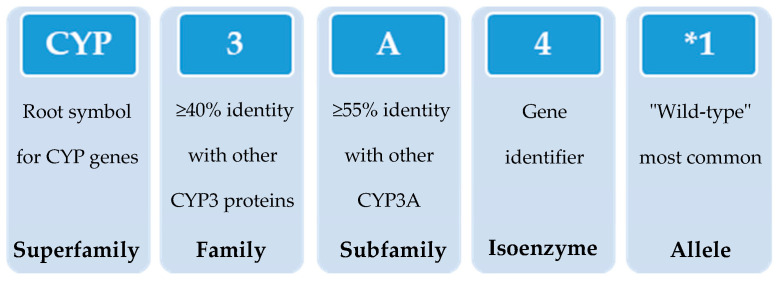
Schematic representation of the nomenclature system of CYP genes (example of CYP3A4*1).

In 1987, the gene superfamily nomenclature system, based on evolutionary divergence of CYP, was proposed [54,83]. Since then, CYPs are organized into families and subfamilies, based on the percentage of amino acid sequence identity (Figure 3) [61,92,93]. Cytochrome P450s forms sharing an identity of ≥40% constitute a particular family designated by an Arabic numeral, whereas enzymes with ≥55% identity are assigned to a particular subfamily designated by a letter. Finally, the gene coding the isoenzyme is designated by an Arabic numeral (https://drnelson.uthsc.edu/) (accessed on 20 June 2021). In humans, the CYP superfamily consists of 57 genes (and 58 pseudo-genes) divided into 18 families and 44 sub-families and can be classified based on major substrate classes (Table 1) (https://cyped.biocatnet.de/) (accessed on 20 June 2021) [5,25,82,94].

## 4. Location and Function of Cytochrome P450s in Mammals

Cytochrome P450s are expressed virtually in all tissues, with highest concentrations found in the small intestine, but particularly in the liver. These membrane-bound hemeproteins contain more than 500 amino acid residues and a single heme prosthetic group in the active site [14,25,26,32]. CYPs are abundant in the microsomal fraction of the liver, playing a central role in bile acid biosynthesis and in the metabolism of foreign compounds [5,25,94]. CYPs are also involved in the homeostasis of steroid hormones, with relevant CYP forms present in the inner membrane of mitochondria of steroidogenic tissues, such as adrenal cortex, testis, ovary, breasts, and placenta [43,44]. Additionally, CYPs are of importance in vitamin metabolism, metabolism of unsaturated fatty acids, and in cholesterol biosynthesis [54,82,95] (Table 1).

The human gut microbiome represents a site for xenobiotic metabolism, altering the pharmacokinetics outcome of drugs, environmental toxicants, and heavy metals. Increased metabolism or biactivation of xenobiotics by the gut microbiome may occur, either through the intestinal tract or re-entering the gut via enterohepatic circulation. This is dependent on the enzymatic activity within the microbial niche [96,97]. CYP host expression in mice showed to be modulated by the collection of microorganisms in the gastrointestinal tract via altered xenobiotic nuclear receptors activity [98,99]. In a dual effect, the microbiota modulates the pharmacokinetics of the xenobiotics, while reciprocally xenobiotics can influence the viability and metabolism of the microbiota.

Cytochrome P450s catalyze a variety of oxidation and reduction reactions involving broad, and in many cases overlapping substrate specificity (Figure 2) [8,18,26,100,101,102,103,104,105,106]. In this context, a single compound can be metabolized by different CYP isoenzymes, in a complex biotransformation enabled by multiple pathways, resulting in numerous metabolites. On the other hand, a unique compound can be metabolized by a single CYP originating different metabolites. The typical CYP-mediated monooxygenation consists of the incorporation of one oxygen atom into the substrate (RH + O_2_ + 2e^−^ + 2H^+^ **^→^** ROH + H_2_O), although the activated oxygen atom may not necessarily be incorporated but used in different types of reactions. Reactions catalyzed by CYPs include: aliphatic, aromatic and *N*-hydroxylation; epoxidation; *N*-, *O*- and *S*-dealkylation; *N*- and *S*-oxidation; oxidative deamination; dehalogenation; dehydrogenation; dehydration; C-C bond cleavage; isomerization; reduction; and esterase [107].

The energy required to activate oxygen is supplied to microsomal CYPs (including those of families 1–4, involved in xenobiotic metabolism, *see below*) by cytochrome P450 oxidoreductase (CPR) [108]. This reductase obtains two electron equivalents in the form of a hydride (H^−^) from NADPH, which is received by its flavin adenine dinucleotide (FAD) moiety (reductase) and subsequently donated to CPR’s second flavin prosthetic group, flavin mononucleotide (FMN) (transporter). Through an extensive open/close protein dynamics, the FMN is reduced (inter-flavin electron transfer, closed conformation) and subsequently, CPR undergoes a large rearrangement, allowing the interaction of CYPs with the FMN domain of CPR (open conformation), with electron equivalents transferred, one at a time, to the heme group of CYPs (inter-protein electron transfer) [80,81,109,110,111,112,113]. Additionally, cytochrome *b*_5_ may play an auxiliary role in sustaining CYPs in their activity, by donating the second electron, which is facultative and dependent on the CYP isoenzyme and/or substrate [75,76,77,78,79].

## 5. The Central Role of Cytochrome P450s as Xenobiotic-Metabolizing Enzymes

In humans, the CYP enzyme family represents the most important enzymatic system involved in Phase I drug metabolism [5,10,13,20,25]. A survey on literature databases of human oxidoreductases and CYP enzymes implicated in the Phase I metabolism evidenced that CYPs are involved in the vast majority (approx. 90–95%) of enzymatic reactions in the metabolism of xenobiotics [7]. Additional enzyme systems may play a role in hepatic Phase I metabolism, albeit to a lesser extent than CYPs, as mentioned above. These include FMOs, NQOs, amine oxidases, alcohol dehydrogenases, esterases and peroxidases [7,13,14,15,16].

Although the majority of the isoenzymes of the 18 human CYP families have specific functions in the metabolism of endobiotics, about 15 isoforms belonging to CYP families 1, 2 and 3 (Table 2) are accountable for 70–80% of all Phase I metabolism of clinically used drugs [25] and are involved in the biotransformation of a vast diversity of environmental chemicals (approx. 90%), including 66% metabolism reactions of chemical carcinogens [114]. From these, CYP1A2, CYP2C9, CYP2D6 and CYP3A4/5, are responsible for about 72% of all CYP-mediated metabolism of clinically marketed drugs [25]. Although typically contributing to the ω-oxidation of endogenous fatty acids and eicosanoids, members of the CYP4 family are additionally involved in xenobiotic metabolism (e.g., CYP4A11, CYP4F2 and CYP4F12), albeit to a much lower extend than isoforms of the CYP1–3 families [115,116,117,118].

The CYP-enzymes involved in xenobiotic metabolism have evolved to protect humans against potential toxic agents. However, CYP-mediated biotransformation may result in metabolic activation of environmental chemicals to reactive carcinogenic products, a process known as “lethal synthesis” or bioactivation [14,25,82,94,114,119,120]. While CYPs may catalyze the activation of procarcinogens to electrophilic ultimate carcinogens, the Phase II enzymes in general detoxify electrophilic intermediates into non-toxic substrates, with few exceptions of non-canonical bioactivation through conjugation reactions [19]. The potentially toxic reactive metabolites that “escape” from Phase II metabolism are able to covalently interact with nucleophilic structures such as those of nucleic acids, proteins, or lipids, causing cell damage and potentially triggering carcinogenesis, teratogenicity, and/or adverse drug reactions (ADRs) [1,3,20,58,103,104,105,114,120] (Figure 1). Several CYP-mediated oxidations contribute to the synthesis of more toxic metabolites, after exposure to specific compounds such as: (**i**) polycyclic aromatic hydrocarbons (PAHs) (e.g., benzo[a]pyrene, Figure 2D), including nitro-polycyclic aromatic hydrocarbons, derived from incomplete combustion; (**ii**) heterocyclic aromatic amines (HAAs) from charbroiled meats; (**iii**) aromatic amines as dyes, or present in pesticides, tobacco smoke and pharmaceuticals (e.g., paracetamol, Figure 2E); (**iv**) nitrosamines present in tobacco smoke (e.g., nitrosamine 4-(methylnitrosamino)-1-(3-pyridyl)-1-butanone, NNK, Figure 2F) and diet, formed from nitrites and nitrates; (**v**) toxins present in food products (e.g., grains or cereals) contaminated with pathogenic microorganisms (e.g., aflatoxin B_1_ produced by *Aspergillus flavus* and *A. parasiticus*, Figure 2G). These xenobiotics are transformed to their toxic forms—usually electrophiles such as epoxides, hydroxylamides, acyl halides, among others—through CYP-mediated activity [5,14,106,107,121].

## 6. Genetic Determinants in Cytochrome P450s Expression and Activity

**Figure 4:**
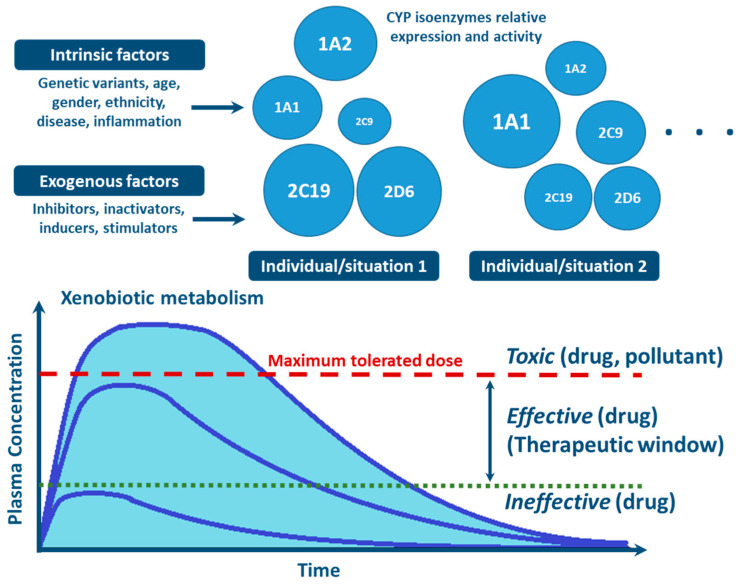
Factors influencing inter- and intra-individual variability in CYP activity and expression, and their impact on xenobiotic metabolism (CYP circles: size indicate the importance of the different inter- and intra-individual levels of expression and function of CYP enzymes).

With the advances in research during the last 30 years, it became clear that the effects of genetic variability of DMEs, particularly those of the CYP enzymes complex, are highly relevant in terms of drug response and detoxification or bioactivation of xenobiotics in general [24,26,57,59,114]. Cytochrome P450s exhibit genetic polymorphisms with multiple allelic variants, demonstrating frequencies varying between different populations and ethnicities [57,58,59,82,106,122] (Figure 4). These include single nucleotide variants, small deletions or insertions, and copy number variants (gene deletion or duplication/amplification, the later more frequent) [55,56,57]. These genetic variants can alter structurally the CYP enzyme or its expression, resulting in normal, reduced, increased or absence of activity [58,59,82,122,123]. A nomenclature system has been set up for the CYP alleles (using the suffixes *1, *2, *3…; where *1 designates the “wild type,” or most common gene-variant) (Figure 3). Allelic variants are summarized and described on the home page of the human CYP allele nomenclature committee, at *Pharmacogene Variation (PharmVar) Consortium* (https://www.pharmvar.org/htdocs/archive/index_original.htm) (accessed on 20 June 2021).

Isoforms CYP2C9, 2C19 and 2D6 present the highest genetic variability in the human population, with so far 70, 38 and 145 allelic variants being identified, respectively (Table 2). These three CYPs have been estimated to account for approximately 35–40% of oxidative drug metabolism and a quarter of biotransformation of xenobiotics in general, including environmental and industrial pollutants [7,17,25,114]. The high frequencies of genetic polymorphism of these three CYPs were demonstrated to cause significant functional effects and high penetrance in individual susceptibility in the human population [4,5,56,57,58,82]. Conversely, severe loss-of-function alleles or functional gene duplications are rare in genes encoding CYP1A2 and 3A4 (21 and 32 allelic variants, respectively) [7,14,25,57,122]. These two CYPs together are responsible for 35% and 30% of drug- and of general chemical metabolism, respectively.

This extensive genetic variability, causing inter-individual differences in expression and activity of human CYPs is considered to be one of the major causes in the lack of efficacy and in ADRs of therapeutic drugs, as well as in the variability of toxic outcome after exposure to environmental compounds [55,56,58,124,125]. Pharmacogenetic testing of CYPs is gaining attention due to the possibility of developing safer drugs and patient-tailored drug therapy in precision medicine [5,25,26]. The occurrence of specific gene variants translates into four major metabolizer phenotypes: (**i**) ultrarapid metabolizers (UM), involving two or more active gene copies or allelic variants, encoding more efficient enzymes or over-expressed CYP isoforms; (**ii**) extensive metabolizers (EM), carriers of two functional CYP alleles; (**iii**) intermediate metabolizers (IM), heterozygous for a defect allele or carrying two alleles causing combined decreased activity of a CYP; (**iv**) poor metabolizers (PM), carrying two defective alleles, producing CYPs with very low or without activity/function [55,56,122,125]. A potential additional genetic variability in CYP mediated xenobiotic metabolism has recently been revealed. This regards the genetic variability of CPR (encoded by the *POR* gene), the obligatory redox partner of microsomal CYPs for the reception of electron equivalents to sustain their activity. Recent data suggest that natural occurring genetic variants of *POR* may lead to altered CYP mediated drug metabolism [126,127,128,129].

Genotyping provides sequence data allowing the estimation of the expectable CYP metabolizer phenotypes. However, additional determinants have been shown to play a role in modulating CYP enzymes function, such as those of the environment and physio-pathological conditions (*discussed below*) [25,130,131]. Of relevance also to mention two main epigenetic mechanisms affecting CYP gene expression: (**i**) altered DNA methylation —involved in biased cellular control of gene expression; and (**ii**) microRNA (miR) regulation—affecting expression levels of target CYPs [5,132,133]. The inhibition of methylation in hepatic cell lines has been reported to induce CYP genes expression, particularly CYP3A genes [134]. Additionally, methylation patterns in the promoters of CYP genes seem to be different in distinct physiological conditions or environmental exposures (e.g., decreased inducibility of CYP1B1 due to promoter methylation at multiple CpG sites; lower methylation in the CYP1A1 promoter found in heavy smokers) [132,135]. Direct regulation of CYPs by miRs was evidenced for CYP1B1 (miR-27b), CYP2C9 (miR-130b), CYP2C9 (miR-34a), CYP2E1 (miR-378), and CYP3A4 (miR-27b, miR148a, and miR34a) [133,136,137,138]. Additionally, histone protein modification, an epigenetic mechanism that may affect chromatin structure, impacting accessibility, have been indicated to be involved in transcriptional regulation of CYP expression. Epigenetic patterns leading to divergent CYP-mediated metabolism are normally reversible, tissue-specific and highly dependent on environmental and individual physio-pathological conditions [25,56,139].

## 7. Nongenetic Factors Influencing Cytochrome P450s Expression and Activity

Factors such as age, sex, hormone levels, and environment, as well as pathological conditions such as infection, inflammation, cholestasis, and cancer are aspects demonstrated to influence CYP expression and activity [25,130,131] (Figure 4). Other biochemical factors such as protein-protein interaction—involving CYP interaction with redox partners and other proteins with allosteric regulatory effect [76,77,81,129], or substrate-substrate interaction—consisting in CYP activity inhibition due to substrate interference/competition mechanism, are also implicated in CYP function [25,100,140,141,142].

**Figure 5:**
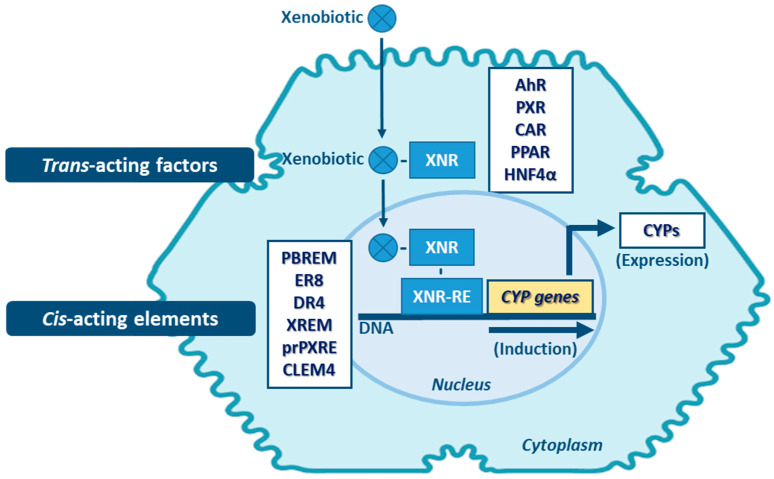
Main mechanisms of CYP induction. AhR: aryl hydrocarbon receptor; CAR: constitutive androstane receptor; CLEM4: constitutive liver enhancer module 4; DR4: direct repeat 4; ER8: estrogen receptor 8; HNF4α: hepatocyte nuclear factor 4α; PBREM: phenobarbital-responsive enhancer module; PPAR: proliferator-activated receptor; prPXRE: proximal PXR responsive element; PXR: pregnane nuclear receptor; XNR: xenobiotic-nuclear receptors; XNR-RE: xenobiotic-nuclear receptors-responsive elements; XREM: xenobiotics-responsive enhancer module. (Note: other regulatory factors and elements not referred may be involved in CYP expression induction).

Transcriptional activation is described as the main process of induction of CYP genes and protein levels [143]. Yet, mRNA and protein stabilization, or inhibition of protein degradation pathways will also lead to altered levels of CYP activity [144]. Proteasome-mediated degradation, phosphorylation, and long non-coding RNA (lncRNAs)-related mechanisms, are among the non-canonical post-transcriptional regulation pathways of CYPs [145,146,147]. Induction of expression of CYP enzymes, and of DMEs in general, involves a complex expression-regulation network dependent on cell-membrane- and nuclear-receptors, promoter-regulation sequences of the cis-acting elements, and of trans-acting activators and repressors, which may be shared among the same enzyme family and between different DME families. Expression of genes encoding CYPs involved in xenobiotic metabolism (mainly of CYP families 1–4) is highly inducible and can be transcriptionally activated by xenobiotics through xenobiotic receptor-dependent mechanisms (Figure 5) [26,148]. Several receptors mediate the induction of these CYPs, such as: the aryl hydrocarbon receptor (AhR)—CYP1 genes; the pregnane nuclear receptor (PXR)—CYP2A6, 2B, 2C, and 3A genes; the constitutive androstane receptor (CAR)—CYP1A, 2A6, 2B, 2C8, 2C9 and 3A4 genes [5,144,148,149,150,151,152,153]. Typically, CYP1A1 and 1A2 are highly inducible by numerous xenobiotics that act as AhR ligands. Genes of these two CYP1A members are arranged in a head-to-head orientation, sharing a common bi-directional promoter with at least 13 AhR response elements [154,155]. Additionally, transactivation of both CYP1A promoters by CAR is also possible through a common cis-regulatory estrogen receptor element (ER8) in the 5′-flanking region [149]. Transcriptional regulation of CYP2A6 gene involves PXR and CAR activators via direct repeat 4 (DR4) elements [150]. In vivo and in vitro studies are indicative that transcriptional upregulation of CYP2A6 may also occurs through an estrogen receptor-dependent pathway [156,157]. CYP2B6 expression is majorly regulated by an orphan CAR (NR113) via a phenobarbital-responsive enhancer module (PBREM), while PXR (NR112) contributes to smaller fraction in CYP2B6 induction through a distal xenobiotics-responsive enhancer module (XREM) [151,152]. The CYP2C genes are variable in their relative inducibility, which is dependent on ligands of the PXR and CAR, glucocorticoid (GR), and vitamin D nuclear receptor (VDR) pathways [158,159]. CYP2C9 is the highest expressed member of this subfamily in the liver, requiring cross-talk between distal PXR and CAR sites and proximal hepatocyte nuclear factor 4α (HNF4α) binding sites in its promoter [160,161]. The proximal PXR responsive element (prPXRE), REM, and the constitutive liver enhancer module 4 (CLEM4) are cis-regulatory elements responsible for inducible transcriptional regulation of CYP3A genes via xenosensors PXR and CAR [153,160,162]. Peroxisome proliferator-activated receptor (PPAR) also contributes to inducible and constitutive regulation of CYP3A4, the major expressed member of the subfamily [163]. Constitutive transcriptional regulators of CYP3A genes include members of CCAAT/enhancer-binding proteins (C/EBP) and HNFs [161,164,165]. Although most of the studies on inducibility of expression of CYP4A subfamily genes have been performed in mouse and rat models, accumulated data are indicative that PPAR mediates induction of expression of members of this subfamily in humans [116]. Expression of the CYP4F2 gene seems to be transactivated by the sterol regulatory element-binding protein (SREBP) and AMP-activated protein kinase (AMPK) activators [118,166].

Inhibition of CYP enzymes impairs the biotransformation of clinically used drugs or environmental compounds, resulting in higher plasma concentrations of these xenobiotics, which may lead to toxicity (Figure 4) [130,144]. On the other end, if the compound is a prodrug, the efficacy of the therapeutic regimen could be decreased as concentrations of the metabolite (the active drug), may fall below effective levels. Inhibitors of CYP enzymes can be classified into three mechanistically distinct groups, namely agents that form (**i**) reversible complexes (competitive or noncompetitive), (**ii**) quasi-irreversible complexes with the heme-iron atom, (**iii**) irreversible complexes through covalent binding to particular residues of the CYP protein. The latter disrupt critical interactions with its redox partners, i.e., CPR or *b*_5,_ or of the heme moiety, accelerating degradation and/or oxidative fragmentation of the prosthetic heme [81,140,141,142]. In competitive reversible inhibition two structurally different molecules transiently compete for the same CYP isoenzyme irrespective of whether these are substrates for the enzyme. In noncompetitive reversible inhibition, a molecule binds to a site other than the active site, causing allosteric modulation of CYP function [5,140].

Dietary factors such as phytochemicals affect CYP expression and activity which may be of importance in diet-drug interactions. Several studies evidenced the inhibitory properties of flavonoids by structural interference with CYP proteins [167]. Additionally, soy components seem to promiscuously modulate several nuclear receptors including AhR, PXR, PPAR and liver X receptor (LXR), altering drug pharmacokinetics and therapeutic efficacy [168] (Figure 5). Other factors have been described to be involved in the induction or inhibition of CYP enzymes, in particularly conditions implying underlying chronic inflammation, such as bacterial, parasitic or viral infections (HIV, hepatitis C), sepsis, rheumatoid arthritis, liver transplant, multiple myeloma, chronic liver disease and cancer [130,131]. Proinflammatory states with secretion of large amounts of cytokines seem to be implicated in divergent xenobiotic metabolism. Cytokines such as interleukins IL-1β, IL-2, IL-4, IL-6, IL-10, and IL-23, interferon gamma (IFNγ), transforming growth factor beta (TGFβ), tumor necrosis factor alpha (TNFα), but also factors involved in infection such as lipopolysaccharides (LPS), have been reported to directly or indirectly modulate CYP expression, as demonstrated in hepatic cell lines, animal models and humans (e.g., patients with cancer undergoing immunotherapy) [130,131,142,169,170,171,172,173,174]. These inflammation factors generally exert a down-regulation of CYP expression, although in some cases, the reverse was noted. The importance of a particular cytokine in CYP regulation will depend on many factors, including its concentration in the liver, particularly in the vicinity of the hepatocytes, time course of its production, modulation by other cytokines, and concentrations of natural antagonists (e.g., IL-1-ra) and facilitators (e.g., specific soluble receptors) [25,131,174]. Cross-talks between cytokine levels and xenobiotic nuclear receptors (i.e., PXR and CAR) have been reported to contribute to the modulation of the CYP activity. During inflammation, nuclear factor κB (NF-κB) represses both PXR and CYP expression through protein-protein interactions with the PXR pathway. Upregulation of protein kinase C (PKC) and cAMP-dependent protein kinase A (PKA) seem to be involved in the repression of CYP expression associated with liver inflammation [146,175]. Moreover, extra-hepatic conditions (e.g., infection, tumors) involving inflammation also reduce the capacity of hepatic drug metabolism due to downregulation of hepatic CYP expression, probably mediated via inflammatory cytokines released by remotely inflamed organs, reaching the liver via systemic circulation [176,177].

## 8. Final Remarks

This short review is intended to highlight main aspects of the central role of CYP enzymes in xenobiotic metabolism, of high significance in clinical pharmacology and toxicology. Although with over seven decades of research and major scientific breakthroughs, several questions and challenges still maintain regarding CYP-mediated metabolism. Current research is mainly focused on obtaining more detailed and precise knowledge regarding: (**i**) functional properties and differences of CYP isoenzymes; (**ii**) their interspecies functional variance; (**iii**) tissue distribution and cellular location; (**iv**) regulatory mechanisms of gene expression; (**v**) population genetic and epigenetic determinants and variability; (**vi**) physio-pathological and environmental factors influencing expression and activity; (**vii**) genotype–phenotype correlation; (**viii**) and overall clinical impact. The scientific literature cited in this short-review, and many more studies not referred, are evidence of the remarkable efforts and achievements in understanding CYPs as the central Phase I enzyme family responsible for the oxidative biotransformation of xenobiotics, integrated in a vast and complex physiological detoxification network.

## Figures and Tables

**Table 1:** Main classes of compounds metabolized by CYPs and major isoenzymes involved their biotransformation.

Classes of Compounds	CYP Isoenzymes
**Sterols**	**1B1** **, 7A1, 7B1, 8B1, ** **11A1** **, ** **11B1** **, ** **11B2** **, 17A1, 19A1, 21A2, ** **27A1** **, 39A1, 46A1, 51A1**
**Xenobiotics**	**1A1** **, 1A2, 2A6, 2A13, 2B6, 2C8, 2C9, 2C18, 2C19, 2D6, 2E1, 2F1, 3A4, 3A5, 3A7**
**Fatty acids**	**2J2** **, 2U1, 4A11, 4B1, 4F11, 4F12, 4F22, 4V2, 4X1, 4Z1**
**Eicosanoids**	**4F2** **, 4F3, 4F8, 5A1, 8A1**
**Vitamins**	**2R1** **, ** **24A1** **, 26A1, 26B1, 26C1, ** **27B1** **, ** **27C1**
**Unknown**	**2A7** **, 2S1, ** **2W1** **, 4A22, 20A1**

Microsomal CYPs (CPR as obligatory electron donor) in **black** and mitochondrial CYPs (adrenodoxin/adrenodoxin reductase as obligatory electron donor) in **green**.

**Table 2:** Genetic variability and importance of the main CYP isoenzymes involved the metabolism of xenobiotics.

CYCYP Isoenzyme	Polymorphism Frequency	Functional Effects	Allelic Variants	Participation in the Metabolism of Xenobiotics
**1A1**	Relatively high	Rare	13	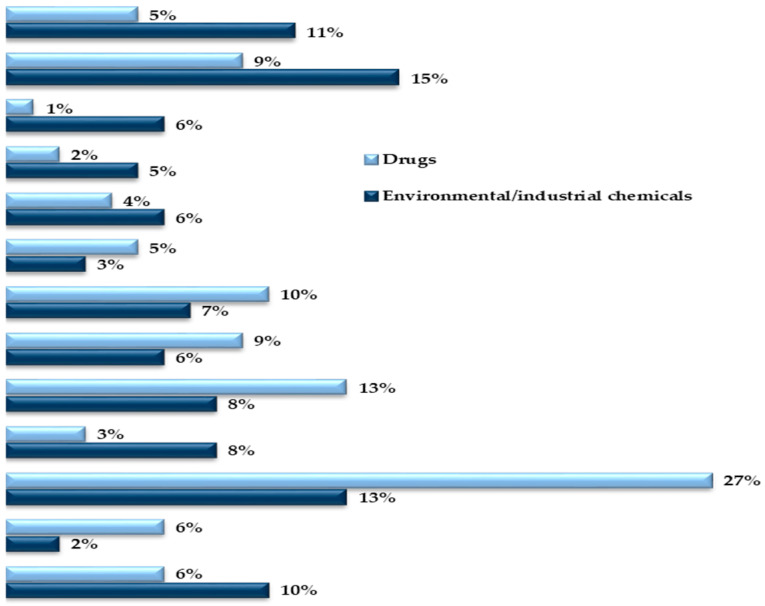
**1A2**	High	Rare	21	
**1B1**	Frequent missense mutations	Rare	26	
**2A6**	Higher in Orientals than in Caucasians	Significant	45	
**2B6**	High	Significant	38	
**2C8**	High	Significant	14	
**2C9**	Relatively rare in Caucasians	Very significant	70	
**2C19**	High	Very significant	38	
**2D6**	Very High	Highly significant	145	
**2E1**	Low	No significance	7	
**3A4**	Low	Low significance	32	
**3A5**	High	Significant	9	
**Other ^(a)^**	-	-	-	

**^(a)^** Other CYP isoenzymes involved in smaller fraction of xenobiotic metabolism (2A13, 2C18, 2F1, 2J2, 3A7 and some members of the CYP4 family). Due to substrate overlapping specificity, CYP1B1 (typical sterols metabolizer) and 2J2 (typical fatty acids metabolizer) were also included in the CYP xenobiotic metabolizer analysis.

## Data Availability

Not applicable.
